# Population homogeneity with unequal exploitation and recruitment contribution within the 2700 km geographic distribution of the commercial hairy crab *Romaleon setosum* (Decapoda: Cancridae) in Chile

**DOI:** 10.1371/journal.pone.0336291

**Published:** 2025-11-10

**Authors:** Branco Tubin-Arenas, Noemi Rojas-Hernández, Ciro Rico, Caren Vega-Retter, Luis Miguel Pardo, David Veliz

**Affiliations:** 1 Departamento de Ciencias Ecológicas, Facultad de Ciencias, Universidad de Chile, Santiago, Chile; 2 Centro de Ecología y Manejo Sustentable de Islas Oceánicas (ESMOI), Universidad Católica del Norte, Coquimbo, Chile; 3 Instituto de Ciencias Marinas de Andalucía (ICMAN), Consejo Superior de Investigaciones Científicas (CSIC), Campus Universitario Río San Pedro, Cádiz, España; 4 Instituto de Ciencias Marinas y Limnológicas, (ICML), Laboratorio Costero Calfuco, Facultad de Ciencias, Universidad Austral de Chile, Valdivia, Chile; 5 Centro FONDAP de Investigación de Dinámica de Ecosistemas Marinos de Altas Latitudes (IDEAL), Valdivia, Chile; Sao Paulo State University Julio de Mesquita Filho: Universidade Estadual Paulista Julio de Mesquita Filho, BRAZIL

## Abstract

Knowledge of the genetic structure of commercially exploited marine species populations is crucial to gaining insights into stock dynamics and population connectivity. This information is essential for effective fisheries management. Economically important benthic fishery species include brachyuran crabs, with the hairy crab *Romaleon setosum* being an important resource in Chile and Peru. The exploitation is uneven along the Chilean coast, with two areas accounting for 67% of the total biomass caught per year. The genetic structure of the *R. setosum* population along the Chilean coast was analysed based on the variability of thousands of single-nucleotide polymorphisms (SNPs) in individuals from 10 areas covering 2700 km of coastline. After data filtering, 256 individuals and 2,383 SNPs remained. There was no evidence of genetic population structure within the studied area, suggesting that there is only one genetic population of *R. setosum* along the coast of Chile. Therefore, among Cancridae species, *R. setosum* has the largest geographic distribution of a single population described worldwide to date. Finally, the analysis of gene flow showed that the zone with the highest fishery also provided the highest proportion of migrants to the other zones. This study emphasises the urgency of local management of source populations in conjunction with the use of size and sex for fisheries control of *R. setosum* along the Chilean coast.

## Introduction

The adults of most benthic marine organisms have low mobility, and the planktonic larval stage facilitates connectivity among populations in different geographical locations. Depending on the species, these larvae can remain in the water column for minutes, months, or even years [1]. Generally, species with longer planktonic larval stages can travel greater distances and maintain low genetic population structure [2].

Ocean currents and larval behaviour, among other factors, also influence the displacement of planktonic larvae in the water column, despite the duration of their development being one of the crucial factors influencing population connectivity [3]. In terms of ocean currents, small-scale physical processes (e.g., turbulence and small-scale eddies), medium-scale features (meanders), and large-scale phenomena (main currents) have been linked to larval displacement [4–6]. In terms of behaviour, vertical movements (ontogenetic or day-night) may alter dispersal patterns and contribute to larval retention near the coast [7,8].

In addition, in species with a wide geographical distribution, environmental discontinuities can lead to genetic structure or reduce connectivity among populations in different biogeographic regions. Studies on different species have shown that these environmental breaks affect the genetic connectivity of species with a short (<one month) planktonic developmental period, but not of those with longer durations [6,9,10]. For example, genetic differences were described for fishes located on opposite sides of the Caribbean Sea [11], for invertebrates on the North and South islands of New Zealand [12], and for crabs located in Northern and Southern Indonesia [13]. Thus, there is evidence that various factors may be involved in genetically structuring populations within a species’ geographic distribution.

The expansion of fisheries necessitates the implementation of protective measures and resource management strategies tailored to the ecological requirements of each species [14]. The identification of fishery stocks, which facilitates the application of differentiated management approaches based on the distinct population dynamics and reproductive features of each stock, is a critical consideration [15]. In this context, genetic variability has become a key tool for delineating fishery stocks [16], helping to prevent population depletion when genetically distinct stocks are managed as a single unit [17]. Crustaceans account for around 5% of worldwide total fisheries production, with the shrimp, lobster, and crab industries being the most important [18]. In recent decades, crustacean landings have increased faster than those of other seafood species [19], leading to population declines and affecting ecosystem processes that regulate the trophic cascades [20]. Therefore, a better understanding of gene flow and genetic diversity in commercially exploited species is crucial for developing effective management plans [21,22]. Moreover, understanding migration pathways helps identify source populations that sustain fisheries, and genetic approaches have been applied to assess connectivity, stock identification and sustainability [23,24].

**Fig 1:**
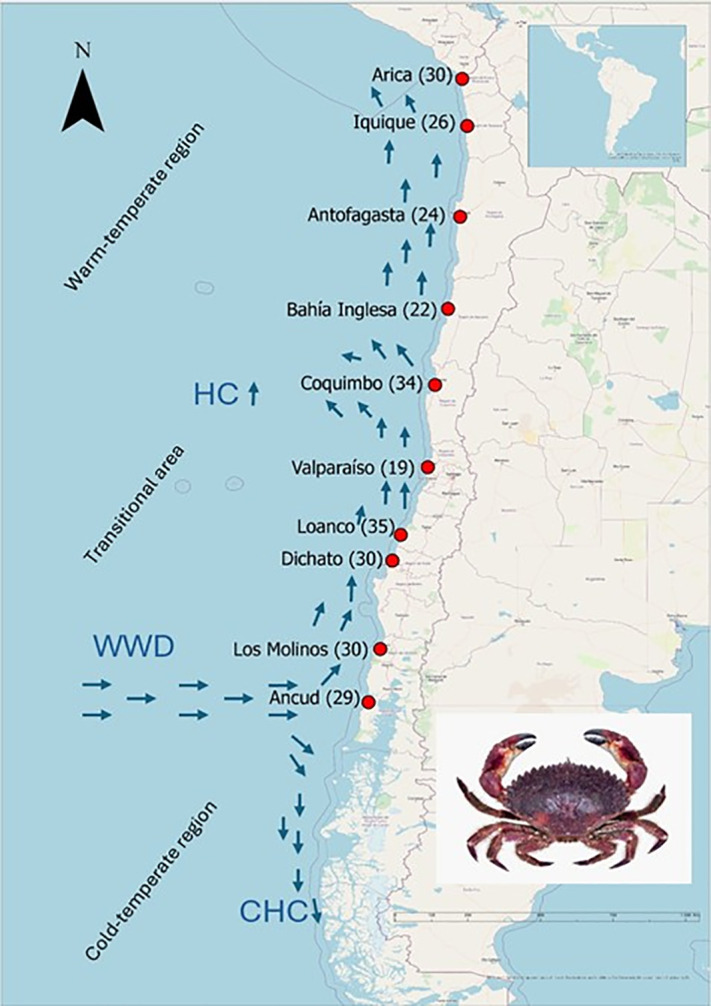
Sampling sites of *R. setosum* in Chile. The red dots represent the sampling sites, with the number of samples given in brackets. WWD = West Wind Drift, HC = Humbolt Current, CHC = Cape Horn Current. The map was drawn with ArcGIS software using the OpenStreetMap (OSM) basemap shapefile of the ArcGIS software. A photo of *R. setosum* appears in this map. Photo by LM Pardo.

In Chile, three biogeographic areas may shape the population genetic structure and connectivity of marine populations due to their environmental conditions: a warm-temperate region (6–30°S), a cold-temperate region (42–56°S), and a transitional area between them (30–41°S) [25]. These zones are mainly defined by permanent upwelling (interruption at 30°S) and the influence of the West Wind Current (interruption at 41°S) (Fig 1), and they also differ in mean annual temperatures [26]. Within this context, benthic species often show different levels of population discontinuity [10], and species distributed across the biogeographical zones may exhibit genetic and morphological variations among populations. This is the case of the gastropod *Acanthina monodon* [27], *Scurra* limpets [28,29] and the bivalve *Perumytilus purpuratus* [30,31]. Understanding population structure and connectivity across biogeographic zones is critical for fisheries management, as it informs the identification of distinct stocks and their potential resilience to exploitation. Nevertheless, the mechanisms driving the distribution and genetic structure of benthic species in these environments require further research.

In this study, we investigate the genetic population structure of the crab *Romaleon setosum* [32] in Chile, a species belonging to the family Cancridae [33]. The distribution of this species spans from Guayaquil, Ecuador, to the Taitao Peninsula in Chile [34], covering approximately 5000 km of coastline. *Romaleon setosum* has a planktonic larval stage that lasts 55–60 days. It reaches its final zoea V stage at a temperature of 13.5°C–14.6°C [35], and its reproduction characteristics and cycle vary with latitude [36]. Eggs are larger in the South, and reproduction shifts from multiple annual cycles in the North to one annual cycle in the South, suggesting some degree of adaptation to temperature [37]. *Romaleon setosum* feeds on other crustaceans, bivalves, and gastropods [38]. It is also part of the diet of coastal fish such as the common blenny (*Paralabrax humeralis*), the rock cod (*Pinguipes chilensis*) [39], and the cephalopod *Robsonella fontaniana* [40]. It is considered one of the most important predators in subtidal environments in northern and central Chile [41–43] as well as one of the most exploited crustaceans in the Humboldt Current [44]. Chile annually lands 218 tonnes per year for human consumption, which is spatially heterogeneous, ranging from 1 ton at 18°S to 109 tons at 35°S [45]. Artisanal fishermen using traps or SCUBA diving harvest the species. To protect this resource, Chilean law provides two measures: a minimum catch size of 12 cm and a ban on catching egg-laying females [46]. The genetic structure of the population of this species in Chile has not yet been fully clarified; a study demonstrated some degree of geographical structure when using allozymes but no difference when using amplified fragment length polymorphisms (AFLPs) [47]. Importantly, this work examined genetic differentiation among populations in a geographical context, without testing for associations with biogeographical regions. Therefore, other genetic markers with better resolution must be used in the detection of genetic population structure, such as single nucleotide polymorphisms (SNPs) [48,49].

This study uses SNPs to investigate the genetic population structure and gene flow of *R. setosum* across geographically diverse locations in Chile. We tested the hypothesis that biogeographic areas influence population genetic structure in this species, predicting that populations separated by biogeographic boundaries would show greater genetic differentiation than populations within the same zone. First, we analysed genetic structure throughout the species’ distribution (~2700 km of coastline, spanning three biogeographic zones) to determine whether biogeographic areas shape population genetic structure. Second, we compared the spatial extent of these genetic populations with patterns reported for other decapod species worldwide, to evaluate whether the scales of population connectivity observed in *R. setosum* are consistent with general trends in marine crustaceans. Finally, we estimated gene flow and related it to regional fishery catches, providing insights into how connectivity among sites supports local fisheries and identifying zones that may function as sources or sinks for these populations.

## Materials and methods

### Sampling sites

In this study, 279 individuals of *R. setosum* were collected from ten locations along the Chilean coast (Fig 1). Specimens were collected in Arica (n = 30), Iquique (n = 26), Antofagasta (n = 24), Bahía Inglesa (n = 22), Coquimbo (n = 34), Valparaíso (n = 19), Loanco (n = 35), Dichato (n = 30), Los Molinos (n = 30) and Ancud (n = 29), corresponding to a coastline of approximately 2700 km between 18°S and 42°S. Local fishermen collected individual crabs by hookah diving and a small muscle sample from a pereiopod was taken from each specimen. The muscle samples were stored and preserved in 95% alcohol for subsequent genetic analysis.

### Genetic analysis

The muscle samples were placed in a 96-well polymerase chain reaction (PCR) plate containing 100 µL of 99% ethanol. DNA extraction and massive parallel DNA sequencing were performed by Dart Diversity Arrays Technology Pty Ltd (DArT; Canberra, Australia). Following the method described by Kilian et al. [50], each DNA sample was digested with restriction enzymes (*PstI* and *HpaII*) and fragments of > 200 base pairs (bp) were ligated with an 8-bp barcode after PCR amplification. PCR products were standardized and sequenced using the Illumina HiSeq 2500 platform (San Diego, CA, USA).

### Quality control and SNP calling

DArT performed demultiplexing, barcode removal, and sequence alignments [50]. The sequence alignment was de novo due to the lack of a reference genome. The SNP database provided by DArT was filtered using the dartR library to retain only highly informative SNPs [51], which are implemented in the statistical software R [52]. The filters were applied to remove: (i) identical cone ID (duplicate SNPs), (ii) SNPs with a read depth < 5 or > 150, (iii) a call rate <99%, (iv) monomorphic loci, (v) SNPs and individuals with > 15% and > 20% missing data, respectively, and (vii) SNPs with a minimum allele frequency (MAF) of < 1%.

To ensure robust and reliable population genetic analyses, we applied a series of quality control procedures. First, the gl.grm function in dartR library was used to detect related individuals in the sample. This approach relies on the Genomic Relationship Matrix (GRM) described by Endelman and Jannick [53]. To identify loci potentially under selection, we applied outflank function implemented in the dartR library; no outliers were detected. SNPs showing significant deviation from Hardy–Weinberg equilibrium were removed for each sampling site, with a false discovery rate (FDR) correction applied at α = 0.05. Finally, linkage disequilibrium (LD) was assessed using PLINK 2.0 software [54]. One SNP from each pair where LD > 0.5 was removed. Although a reference genome for *R. setosum* is unavailable, this conservative threshold minimizes the inclusion of linked loci.

### Genetic diversity and structure

Using the filtered SNP dataset, the expected heterozygosity (H_e_), observed heterozygosity (H_o_), and inbreeding coefficient (*F*_IS_) for the ten sampling sites were estimated using the dartR library. Further, the effective population size (NE) was estimated using the linkage disequilibrium method implemented in the NeEstimator software [55].

We used four methods to describe the genetic population structure of *R. setosum*. First, a principal coordinate analysis (PCoA) was performed to qualitatively analyse the distribution of individuals in a multivariate space. This analysis was performed using the adegenet library [56], which is implemented in the statistical software R. Second, the fixation index (F_ST_) for pairs of sites and its respective probability value (1000 permutations) were estimated using the gl.fst.pop function implemented in the dartR library. P-values for were adjusted from multiple comparisons using the False Discovery Rate (FDR) implemented in the function p-adjust of the R software. Third, the most probable number of genetic clusters (K) was estimated using the Bayesian approach implemented in the ParallelStructure software [57], an extension for a multi-core computation of the STRUCTURE software [58]. We repeated the procedure ten times for each K (K = 1–10), with a burn-in of 100,000 iterations and an after-burn of 1,000,000 iterations. This analysis was conducted at Cipres (Cyberinfrastructure for Phylogenetic Research, UC San Diego, USA). The most likely number of clusters was determined by assessing the probability of each K using the method described in the STRUCTURE software manual [59].

Finally, given the distribution of *R. setosum* across Chile’s three biogeographical regions, we investigated the potential impact of these interruptions on the genetic geographical structure. We performed a nested analysis of molecular variance (AMOVA), nesting the locations within the North, Central, and South regions. We separated the North and Central regions at 30°S, followed by the Central and South regions at 41°S. Consequently, the North region included five sites (Arica, Iquique, Antofagasta, Bahía Inglesa, and Coquimbo), the Central region included four sites (Valparaíso, Loanco, Dichato, and Los Molinos), and the South region included one site (Ancud). This analysis was performed using the poppr.amova function of the poppr package [60] implemented in the statistical software R; statistical significance was estimated with 1000 permutations.

### Gene flow among sampling sites

We used a Mantel test to estimate the potential existence of isolation by distance (IBD) in order to detect a possible equilibrium between migration and drift. This analysis considered the calculated pairwise F_ST_ values and the geographical distance between the sampled sites measured using Google Earth (https://earth.google.com). A Mantel test was performed using GENETIX software [61] and statistical significance was assessed at 10,000 permutations. The Estimating Effective Migration Surfaces (EEMS) programme [62] was used to visualise the global pattern of gene flow among sampling sites. EEMS estimates migration rates to match observed and expected genetic differences within an idealised stepping-stone model. These estimates are then interpolated between sampling sites to visually represent genetic variation and highlight regions of historical gene flow that are above or below average programme [62]. The analysis considered 500 demes and three independent chains with five million Markov chain Monte Carlo iterations, with a burn-in of one million iterations and sampling every 9,999 iterations. The proposed variances were adjusted, considering an acceptance rate of 10% to 40%. The results were visualised using the rEEMSplots package programme [62] implemented in the statistical software R.

We checked the current gene flow between two sampled sites using the BayesAss3-SNPs software [63,64]. We did a burn-in of 200,000 iterations and then another 200,000 iterations after the burn-in, with samples every 100 iterations. We assigned values of 0.2, 0.4, and 0.15 to the mixing parameters (migration rates, allele frequencies, and inbreeding coefficients), respectively. These values were assigned after multiple runs to adjust the acceptance rates to between 20% and 60%, as recommended in the software manual. We carried out five independent runs with different initial seeds to ensure consistency. We compared the percentages of immigrants at each site with the biomass of *R. setosum* from Chile’s political regions to identify the most significant sites in the system. The tons of crabs harvested were obtained from the National Fisheries Service website, which contains information from 2018 to 2021 [45].

### Comparison of the geographic extent of decapod crab populations

We conducted a literature search to compare the geographical extent of our single panmictic population of *R. setosum* with that of other decapod panmictic populations worldwide, as our results indicated. In October 2023, we conducted a targeted search of the Web of Science using the keywords “Decapoda and population genetics” and “crab and population genetics.” We selected articles reporting population genetic analyses using only microsatellites, or SNPs, among the identified ones. We excluded analyses using mitochondrial DNA due to their lower statistical power in detecting genetic population structure using this marker. We used the geographic distances provided in the individual articles. However, in the absence of such information, we measured the distance between sites using Google Earth (https://earth.google.com), assuming linear distances. We created an approximate population distribution map using ArcGIS Pro (www.esri.cl) based on this information to visually compare the geographic distribution of the different decapod species examined in this study and other studies.

## Results

Massive parallel DNA sequencing revealed 46,137 SNPs among the 279 individuals analyzed. After filtering, we retained 256 individuals with 2,383 SNPs. Two individuals from Coquimbo and two from Iquique showed a high degree of relatedness (GRM = 1.09 and 1.36, respectively). Therefore, we excluded one individual from each related pair from further analyses. We removed two SNPs showing significant deviation from the Hardy-Weinberg equilibrium, and one SNP was also removed due to the LD > 0.5.

### Genetic diversity and structure

Three genetic diversity indices were estimated, namely: expected heterozygosity (H_e_), observed heterozygosity (H_o_), and inbreeding coefficient (*F*_IS_); all of them showed similar values at all sites studied (Table 1), with no obvious clinal variation associated with latitude. The H_o_ ranged from 0.0392 in Loanco to 0.0523 in Coquimbo. The inbreeding coefficient (*F*_IS_) varied from 0.1859 in Coquimbo to 0.3516 in Loanco. The N_E_ ranged from 92.3 (8401–99.4) in Arica to infinite values in the southernmost localities (Dichato, Los Molinos and Ancud, Table 1). Pooling all data, the N_E_ = 3463 (2444.1–5876.6).

**Table 1:** Summary of SNPs data of the crab *R. setosum* including sampling sites, geographical coordinates, sample size before (N initial) and after filtering (N after filtering), observed heterozygosity (H_o_), expected heterozygosity (H_e_), F_*IS*_ and Effective Population Size (N_E_) at each study site.

Sampling Site	Coordinates	N initial	N after filtering	H_o_	H_e_	F_*IS*_	N_E_ (95% Confidence Intervale)
Arica	18°28′ S; 70°19′ W	30	28	0.0539	0.0675	0.2167	92.3 (84.1–99.4)
Iquique	20°12′ S; 70°09′ W	26	23	0.0480	0.0617	0.2389	486.5 (293.3–1336.2)
Antofagasta	23°38′ S; 70°23′ W	24	23	0.0496	0.0631	0.2313	668.6 (331.8–3769.8)
Bahía Inglesa	27°07′ S; 70°52′ W	22	18	0.0481	0.0599	0.2203	396.1 (239.9–688.2)
Coquimbo	29°57′ S; 71°20′ W	34	31	0.0523	0.0632	0.1858	1225.8 (568 – Inf)
Valparaiso	33° 01′ S; 71° 39′ W	19	18	0.0421	0.0575	0.2889	Inf (953.6 – Inf)
Loanco	35° 35′ S; 72° 38′ W	35	29	0.0392	0.0593	0.3516	349.9 (255.8–552.9)
Dichato	36° 31′ S; 72° 57′ W	30	30	0.0503	0.0617	0.1990	Inf (2508.8 – Inf)
Los Molinos	39° 51′ S; 73° 23′ W	30	30	0.0484	0.0611	0.2203	Inf (3210.9 – Inf)
Ancud	41° 50′ S; 73° 51′ W	29	26	0.0458	0.0599	0.2508	Inf (922.1 – Inf)

**Fig 2:**
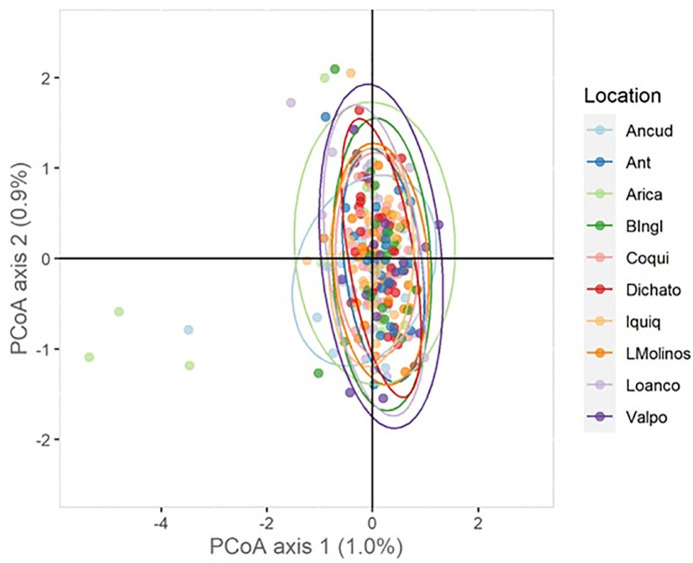
Principal Coordinate Analysis (PCoA) of *R. setosum.* The first and second principal components (*x*-axis and *y*-axis, respectively) explain 1.9% of the total variance.

**Fig 3:**
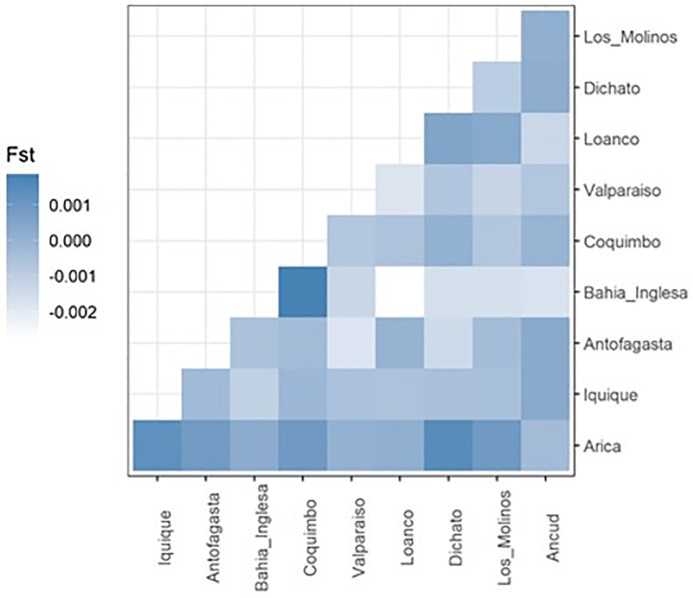
Heatmap of F_ST_ values for pairs of sample sites. No statistical significance was detected in any paired comparison.

**Fig 4:**
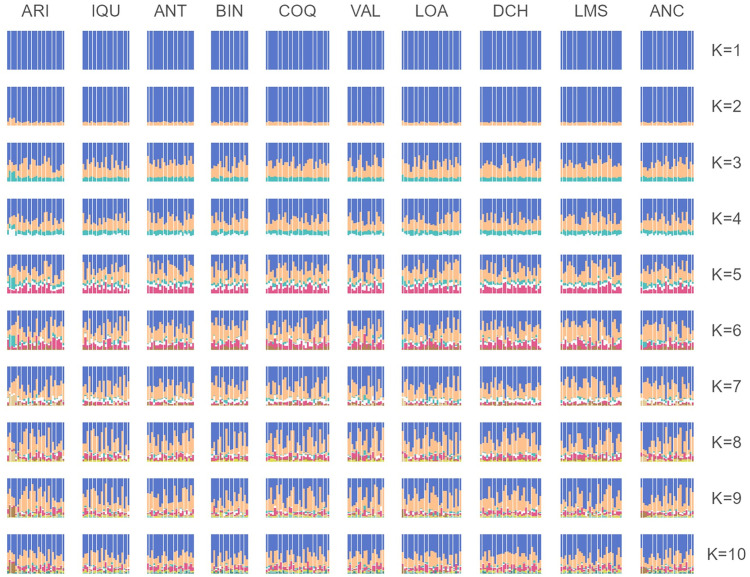
The population structure of *R. setosum* inferred using the STRUCTURE software. *K* = 1 to *K* = 10 was examined using 256 individuals from 10 sampling sites along the geographical distribution of the species in Chile. Each vertical bar represents one individual, and each color represents the probability of belonging to one of the *K* genetic clusters.

According to the four approaches used, there is only one genetic population in Chile’s entire geographical distribution. The PCoA showed that individuals from all sites overlapped in the first two components and explained < 2% of the total variance (Fig 2). Three individuals from Arica (an extreme north sampling site) and one from Ancud (an extreme south sampling site) showed slight differences with whole samples in the multivariate space. The F_ST_ index revealed no significant differences among all pairs of sites (Fig 3 and S1 Table). The Bayesian approach showed that K = 3 was the most likely number of clusters based on the highest lnP(K) value (third plot in Fig 4). However, the ancestry proportions were nearly identical across all individuals with K = 3, showing no evidence of geographical differentiation. This result suggests that, although the algorithm identifies three clusters (as expected under the methodological limitations of the method [65]), these do not represent distinct populations but rather a shared ancestral gene pool, consistent with the absence of contemporary population structure in *R. setosum* along the Chilean coast.

Finally, the AMOVA was performed to detect possible effects of geographical breaks on genetic variance partitioning and revealed no significant differences for regions (p = 0.932) or locations within regions (p = 0.104; Table 2). This analysis also showed low Phi values for both regions (Phi CT = −9.2377 × 10^−4^) and sites within regions (Phi SC = −9.0119 × 10^−4^).

**Table 2:** Results of the Analysis of Molecular Variance (AMOVA) conducted to detect the effect of regions (north, central, south) and localities within regions in the partition of the total variance. The analysis did not detect a significant effect in both components.

Component of the variance	Sigma p	% Variance	P-value
Region	−0.060630	−0.000923	0.9321
Sites within regions	0.059202	0.000902	0.1049
Error	65.633619	>0.999999	

### Gene flow

**Fig 5:**
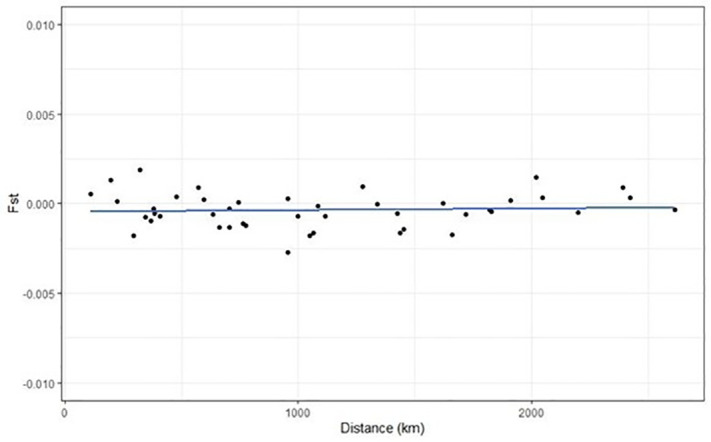
The relationship between geographic distance and F_ST_ among pairs of sampling sites. The Mantel test did not detect a significant association between geographic distance and F_ST_ (*Z* = −32.42, *p* = 0.410).

**Fig 6:**
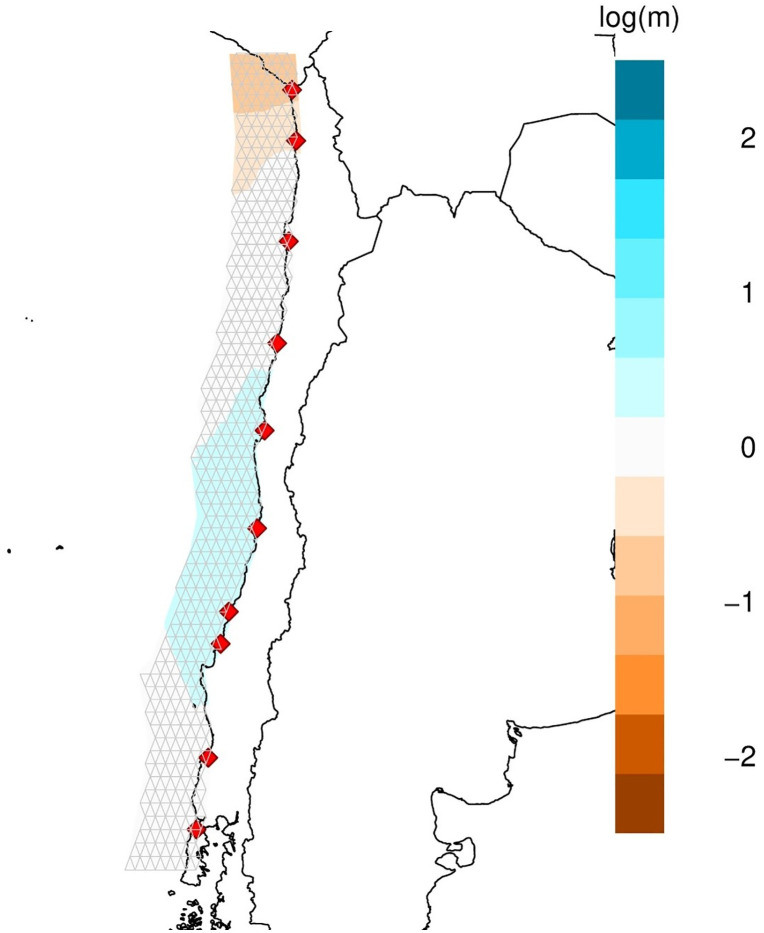
The effective migration rate among the 10 sampling sites. The EMMS software ^82^ estimated the effective migration rate considering the 10 sampling sites. Log(*m*) represents the effective migration rate on a relative log_10_ scale, considering the overall migration rate across the covered area. Blue indicates areas where the effective migration is higher than average, and brown indicates areas where the effective migration is lower than average. The maps were created using the rEEMSplots library implemented in the R statistical software.

A Mantel test was performed to test link between F_ST_ and the distance between two sites, but no relationship was found (Z = −32.42, p = 0.410; Fig 5), which means that migration and drift are not balanced. The migration rate was estimated with the Effective Migration Surfaces (EEMS) program, showing log(*m*) values ≥0 across the entire geographical distribution of *R. setosum* in Chile (Fig 6), with higher values in the intermediate zone of the distribution (Coquimbo to Dichato). Because this species did not exhibit an isolation by distance (IBD) pattern, these analyses suggest that gene flow occurs among all sites with higher values in the middle of Chile’s species distribution.

**Fig 7:**
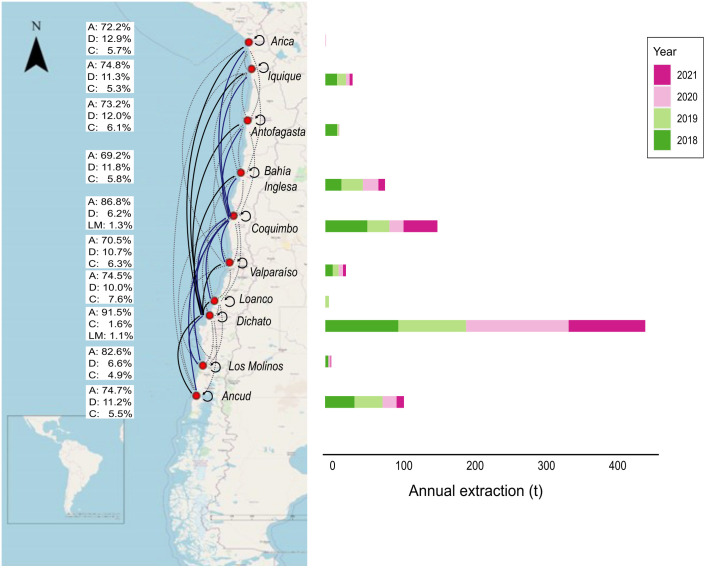
Comparison between migration rates and exploitation levels. Left: Reciprocal migration between pairs of sampling sites. Black arrows represent values of >10% of immigrants at a site, blue arrows represent values of >5% and <10% of immigrants at a site, and dotted arrows represent values of <5% of immigrants at a site. For each sampling site, the figure shows the auto recruitment rate (A) and the first and second ranked sites contributing immigrants (D: Dichato; C: Coquimbo; LM: Los Molinos). Right: Annual extraction (in tons) of *R. setosum* from each region. Dichato and Coquimbo ranked first and second, respectively, for both immigrant rate and tons of *R. setosum* extracted. The map was drawn with ArcGIS software using the OpenStreetMap (OSM) basemap shapefile of the ArcGIS software.

Finally, the current gene flow estimated with the BayesAss3-SNPs software showed an exchange of individuals among all sites, with some exchanges being greater than others. All sampling sites received a large proportion of individuals who originated from their own deme (self-recruitment > 70%), followed by immigrants from Dichato (6–10% of immigrants) and Coquimbo (1–8% of immigrants; Fig 7). When one compares this data to the regional catches of *R. setosum*, the sites where immigrants are most common (Dichato and Coquimbo) also have the highest catch biomass of this crab species every year (Fig 7).

## Discussion

Using SNP variability to determine the population genetics of the crab *R. setosum* revealed an apparent lack of genetic population structure along its range in Chile. This analysis suggests that there is only one genetic population of this species across the 2,700 km of sites studied. Interestingly, gene flow among sampling sites is unaffected by the biogeographic barriers to dispersal found across the species’ geographic range. While this result is not unusual, environmental discontinuities often affect dispersal and gene flow in many marine taxa with varying life history traits [66]. Moreover, the assumption that planktonic larval duration predicts the population structure has been questioned, as larval duration often poorly reflects population connectivity, with biogeographic areas and habitat discontinuities playing an important role [67,68]. Re-evaluating this assumption helps clarify what shapes genetic structure in marine systems and points to the need for studies on other species and geographical areas.

Researchers have argued that biogeographic breaks, primarily related to the type and timing of larval development, lead to changes in species composition [69] and discontinuities in the genetic structure of benthic marine species populations [9]. Thus, species with direct development exhibit greater population differentiation [70], some of which are directly associated with biogeographic breaks [27]. In species with planktonic larvae, these geographical breaks are mainly visible in species with a shorter planktonic larval duration (< 30 days). This pattern has been described in different biogeographical zones, including South Africa [71], Chile [10], Italy [72], and the west coast of the USA [9].

Populations of bivalves and gastropods have shown genetic differences across the two geographical breaks described (30°S and 41°S in Chile). For example, Sanchez et al. [27] described an effect of 30°S latitude on the gastropod *Acanthina monodon*, a species with direct larval development [73]. An impact of the 41°S latitude break on the mussel *Mytilus chilensis* [74], a species with a planktonic larval development of < 45 days [75]. Examples of species with genetic populations unaffected by geographical breaks are fish and benthic marine invertebrates with a planktonic larval development of more than two months. In fish species, the latitudinal differences of 30°S and 41°S do not affect the genetic population structure of the spiny mackerel (*Trachurus murphyi*) [76], and the latitudinal difference of 30°S also affects the population of the red spiny eel [77], which could be explained by the high dispersal ability of all stages of its life cycle. For benthic marine invertebrates with limited dispersal of juveniles and adults, studies found that 41°S latitude had no effect on the genetic population structure of the Chilean abalone *Concholepas concholepas* [78] and the Chilean rock crab *M. edwardsii* [79]. Both species have a planktonic developmental stage lasting over 60 days [80,81]. This suggests that the high dispersal ability of the planktonic larvae of these species allows for gene flow and connectivity between populations across a wide geographic range. Thus, environmental gradients observed in the area [25,26], may influence the genetic population structure of benthic marine invertebrates with limited dispersal ability. The impact of dispersal ability on the genetic population structure of various marine species and its implications for management require further research.

In Chile, a study showed different genetic patterns in *R. setosum* depending on the marker type [47]. Allozymes indicated genetic differentiation between some populations, while AFLP did not show significant differences. Thus, all data, including the present study, suggest that there is only one panmictic population of *R. setosum* along Chile’s coast. This is consistent with research on other species that have long larval stages that occur in water, allowing larval dispersal and consequently gene flow. This study indicates that *R. setosum* shows high connectivity and homogeneous genetic diversity along the Chilean coast, without significant genetic structuring. Overall, these results have important management implications, suggesting that efforts should focus on assessing population biomass across the entire Chilean coast as a single management unit. It is important to note that preserving genetic diversity is imperative, since exploitation could erode genetic diversity and the potential to adapt to environmental changes, affecting a long-term sustainable fishery.

Although the data obtained in the present study reveal the presence of a single genetic population, previous biological evidence suggested the existence of distinct populations when comparing localities from northern and southern Chile. For instance, *R. setosum* showed an increase in egg weight with latitude [36], and females reduce their egg-carrying frequency at higher latitudes, from two to three times per year in northern Chile to only once per year in the southernmost areas [37]. This pattern has been interpreted as an adaptive maternal response, in which increased energy investment per egg at lower temperatures supplies incubating larvae with the resources required for their prolonged development in cold conditions [44]. However, when considered in light of our genetic results, this reproductive gradient may represent phenotypic plasticity rather than local adaptation to different latitudinal environments. In other words, the reproductive traits of *R. setosum* may vary in response to environmental conditions rather than reflecting genetically distinct populations. While this remains a hypothesis, it is necessary to test it with experiments, such as common gardens or reciprocal transplant experiments.

### Worldwide population genetic structure of crabs

**Fig 8:**
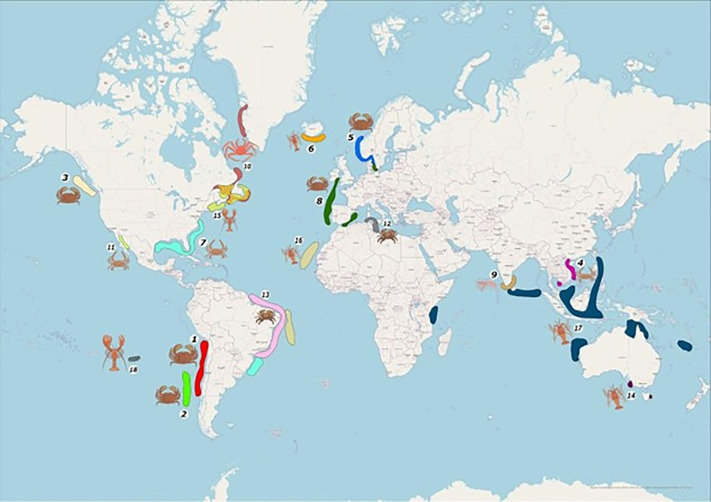
Population geographic distribution of decapods based on the examined articles. Each polygon indicates a genetically differentiated population, and each color corresponds to a different species. The population geographic extension from each article is listed by their position in S2 Table. *R. setosum* from this study ranked first. The map was drawn with ArcGIS software using the OpenStreetMap (OSM) basemap shapefile of the ArcGIS software.

The literature search yielded a total of 106 articles with the key words used, and we selected 18 articles after a qualitative assessment. The species examined in these studies were mainly from the families Cancridae and Palinuridae (S1 Table and Fig 8), and some generalizations arise based on the information gathered. These species typically had only one large population, and those with more than one population were separated by considerable geographical discontinuities, as in the case of the blue crab *Callinectes sapidus* in the USA and Brazil [82]. The species with most geographical extended populations were the lobsters, for example, *Panulirus ornatus* appears to be genetically homogeneous over 6500 km [83]. Within the family Cancridae, *R. setosum* has the largest genetically homogeneous population described (2700 km of distance). It is important to note that our study did not examine the entire geographic range of *R. setosum*, which extends to Ecuador; therefore, the homogeneity of this crab population may extend beyond 2700 km. Finally, the data from all these species suggests that the larvae of decapod crustaceans can disperse long distance enabling connectivity among sampling sites that are several hundreds of kilometres apart.

### Fishery of *R. setosum* and its relationship with reciprocal migration

*Romaleon setosum* is a fishery resource that mainly supplies the local markets. Although *R. setosum* has the third largest landings (178 tonnes/year) among brachyurans after *M. edwardsii* (3645 tonnes/year) and *Cancer porteri* (828 tonnes/year), its fishery along the Chilean coast is not uniform. Between 2018 and 2021, *R. setosum* recorded the highest landings in the Dichato region, with an average of 104 tonnes/year, and in the Coquimbo region, with an average of 46 tonnes/year [45]*.* The gene flow analysis in our study showed that these sites with higher landings also brought the most immigrants to the other sites, suggesting that sites with higher catches contribute more to the immigrants at other sites. A similar pattern was observed in the cephalopod *Octopus mimus* inhabiting northern Chile, where the Antofagasta area exhibited higher landing and emigration rates compared to other sampling sites [84].

To explain this pattern, it is important to note that the Humboldt Current System is categorised as the most productive upwelling worldwide, with areas containing temporal and permanent upwelling in Chile [85], promoting phytoplankton growth through nutrient enrichment and sustaining a large portion of the global fishery [86,87]. In this context, Dichato and Coquimbo have intense and permanent upwelling [88] that increases nutrient concentration and primary productivity close to these areas [89,90]. Thus, this evidence suggests that these sites have the highest increase in nutrients, which increases the biomass of *R. setosum* and, therefore, may produce greater numbers of larvae and recruits. Our results indicate that both sites (Dichato and Coquimbo) are important for the conservation of genetic diversity and population homogeneity of *R. setosum* in Chile. New studies are required to determine the importance of utilising gene flow between local populations with different fishing efforts to ensure the sustainability of this resource over time, as Chile’s crab fishery measures only consider a minimum landing size and a ban on the removal of females.

Monitoring the fishing effort at each site and adjusting regulations accordingly could help maintain a balance between resource availability and utilisation. Although *R. setosum* is not listed as threatened, it is subject to intense artisanal and commercial harvesting, and assessment of abundance and catch remains scarce. Our results show a single genetic population along their distribution in Chile, highlighting that local overexploitation could have consequences for the entire stock. Overall, understanding the impact of gene flow, effective population size, and fishing pressure on *R. setosum* populations will be crucial for the long-term sustainability. By closely monitoring genetic diversity, effective population size, and population structure, scientists can better assess the species’ capacity to maintain adaptive potential in the face of environmental changes and harvesting. From a management perspective, more practical strategies may involve local tailored regulations, such as monitoring fishing effort and biomass at key landing sites (e.g., Coquimbo and Dichato). Additionally, implementing sustainable fishing practices, such as size and catch limits, can help prevent overexploitation and ensure the continued availability of *R. setosum* in the future. Ultimately, a comprehensive approach that integrates both genetic and ecological information will be essential for the successful management of this important marine resource in Chile.

## Supporting information

S1 Table Pairwise FST (above diagonal) and corrected P-value (below diagonal) values for the sample sites of *R. setosum.* (DOCX)

S2 Table Summary of the geographical extension of decapod species populations described with microsatellites or SNPs. The species, family, coordinates, and estimated geographical range are indicated.
(DOCX)
