# Supplementary material for: Population homogeneity with unequal exploitation and recruitment contribution within the 2700 km geographic distribution of the commercial hairy crab Romaleon setosum (Decapoda: Cancridae) in Chile
Source: PLoS One. 2025 Nov 10;20(11):e0336291. doi: 10.1371/journal.pone.0336291 (PMC12599916; doi:10.1371/journal.pone.0336291)
Supplement: S1 Table — (DOCX) [file pone.0336291.s001.docx]

S1 Table. Pairwise F*_ST_* (above diagonal) and corrected P-value (below diagonal) values for the sample sites of *R. setosum*.

|  | Arica | Iquique | Antofagasta | Bahia Inglesa | Coquimbo | Valparaiso | Loanco | Dichato | Los Molinos | Ancud |
| --- | --- | --- | --- | --- | --- | --- | --- | --- | --- | --- |
| Arica |  | 0.00131 | 0.0009 | 0.00027 | 0.00094 | 0.00004 | 0.00016 | 0.00148 | 0.00092 | -0.00032 |
| Iquique | 0.915 |  | -0.00028 | -0.0011 | -0.00016 | -0.00057 | -0.00059 | -0.00047 | -0.00049 | 0.00034 |
| Antofagasta | 0.980 | 0.980 |  | -0.00057 | -0.0003 | -0.00179 | -0.00002 | -0.00142 | -0.00036 | 0.00031 |
| Bahia Inglesa | 0.980 | 0.980 | 0.980 |  | 0.00186 | -0.0013 | -0.00273 | 0.00164 | -0.00164 | -0.00175 |
| Coquimbo | 0.980 | 0.980 | 0.980 | 0.915 |  | -0.00073 | -0.0006 | 0.00007 | -0.00071 | -0.00005 |
| Valparaiso | 0.980 | 0.980 | 0.980 | 0.980 | 0.980 |  | -0.00179 | 0.74 | -0.00123 | -0.00072 |
| Loanco | 0.980 | 0.980 | 0.980 | 0.996 | 0.980 | 0.980 |  | 0.00054 | 0.00037 | -0.00133 |
| Dichato | 0.855 | 0.980 | 0.980 | 0.980 | 0.980 | 0.980 | 0.980 |  | -0.00096 | 0.00021 |
| Los Molinos | 0.980 | 0.980 | 0.980 | 0.980 | 0.980 | 0.980 | 0.980 | 0.980 |  | 0.00013 |
| Ancud | 0.980 | 0.980 | 0.980 | 0.980 | 0.980 | 0.980 | 0.980 | 0.980 | 0.980 |  |
