# Supplementary material for: Population homogeneity with unequal exploitation and recruitment contribution within the 2700 km geographic distribution of the commercial hairy crab Romaleon setosum (Decapoda: Cancridae) in Chile
Source: PLoS One. 2025 Nov 10;20(11):e0336291. doi: 10.1371/journal.pone.0336291 (PMC12599916; doi:10.1371/journal.pone.0336291)
Supplement: S2 Table — The species, family, coordinates, and estimated geographical range are indicated. (DOCX) [file pone.0336291.s002.docx]

S2 Table. Summary of the geographical extension of decapod species populations described with microsatellites or SNPs. The species, family, coordinates, and estimated geographical range are indicated.

| N° | Specie name | Family | Geographical coordinates | Distance approx (km) | References |
| --- | --- | --- | --- | --- | --- |
| 1 | *Romaleon setosum* | Cancridae | 18°46´S 70°33´W - 41°86´S 73°83´W | 2700 | This study. |
| 2 | *Metacarcinus edwardsii* | Cancridae | 33°01′S 71°39´W - 45°26′S 72°55′W | 1700 | Veliz et al. (2022) |
| 3 | *Cancer magister* | Cancridae | 54°15´N 131°55´W - 48°38´N 123°33´W | 960 | Beacham et al. (2008) |
| 4 | *Portunus pelagicus* | Portunidae | a.- 20°99´N 107°35´E - 12°15´N 109°23´E  b.- 10°24´N 103°94´E - 10°00´N 105°07´E | a.- 1150  b.- 150 | Dang et al. (2019) |
| 5 | *Cancer pagurus* | Cancridae | 57°06´N 11°30´E - 62°40´N 06°39´E | 1300 | Ungfors et al. (2009) |
| 6 | *Nephrops norvegicus* | Nephropidae | 64°24´N 23°16´E - 63°40´N 15°48´E | 500 | Pampoulie et al. (2011) |
| 7 | *Callinectes sapidus* | Portunidae | a.- 37°45´N 76°12´W - 26°06´N 97°07´W  b.- 26°90´S 48°64´W - 31°40´S 51°04´W | a.- 4000  b.- 600 | Macedo et al. (2019) |
| 8 | *Carcinus maenas* | Carcinidae | a.- 53°14´N 4°10´W - 36°28´N 6°11´W  b.- 35°34´N 6°00´W - 40°38´N 0°43´W  c.- 58°15´N 11°25´E | a.- 2200  b.- 1000  c.- 1 sampling site | Domingues et al. (2010) |
| 9 | *Aristeus alcocki* | Aristeidae | 09°94´N 76°24´E - 13°06´N 80°29´W | 1000 | Purushothaman et al. (2020) |
| 10 | *Chionoecetes opilio* | Oregoniidae | a.- 69°01´N 52°03´W - 65°36´N 52°49´W  b.- 54°72´N 56°94´W - 45°60´N 60°31´W | a.- 500  b.- 3500 | Puebla et al. (2008) |
| 11 | *Callinectes bellicosus* | Portunidae | 31°30´N 113°56´W - 26°32´N 109°32´W | 700 | Cisneros-Mata et al. (2019) |
| 12 | *Pachygrapsus marmoratus* | Grapsidae | 36°54´N 07°45´E - 32°52´N 13°20´E | 1000 | Deli et al. (2016) |
| 13 | *Ucides cordatus* | Ocypodidae | 02°16´N 50°21´W - 25°73´S 48°44´W | 5000 | Oliveira-Neto et al. (2014) |
| 14 | *Jasus edwardsii* | Palinuridae | a.- 36°33´S 139°24´E  b.- 41°52´S 148°18´E | 2 sampling sites separated by 1000 km | Villacorta-Rath et al. (2017) |
| 15 | *Homarus americanus* | Nephropidae | a.- 50°00´N 55°45´W - 44°84´N 62°22´W  b.- 44°66´N 63°15´W - 41°25´N 69°98´W | a.- 3200  b.- 1200 | Dorant et al. (2022) |
| 16 | *Panulirus echinatus* | Palinuridae | a.- 28°02´N 17°36´W - 16°45´N 24°55´W  b.- 03°51´S 32°25´W - 20°28´S 29°21´W | a.- 1400  b.- 1900 | Gaeta et al. (2019) |
| 17 | *Panulirus ornatus* | Palinuridae | a.- 01°75´S 41°56´E - 10°43´S 40°54´E  b.- 05°87´N 80°59´E - 05°58´N 95°29´E  c.- 11°31´N 109°01´E - 21°87´N 120°82´E - 11°92´N 121°88´E - 04°46´N 118°63´E - 08°58´S 116°06´E  d.- 19°26´S 146°84´E - 10°07´S 142°35´E  e.- 21°95´S 113°91´E  f.- 20°97´S 165°66´E | a.- 950  b.- 1500  c.- 6500  d.- 1000  e.- 1 sampling site  f.- 1 sampling site | Farhadi et al. (2022) |
| 18 | *Panulirus pascuensis* | Palinuridae | 27°16´S 109°33´W - 26°47´S 105°36´W | 400 | Meerhoff et al. (2018) |
